# Unanswered clinical questions in the management of cardiometabolic risk in the elderly: a statement of the Spanish society of internal medicine

**DOI:** 10.1186/1471-2261-14-193

**Published:** 2014-12-18

**Authors:** Ricardo Gómez-Huelgas, Vicente Giner-Galvañ, José M Mostaza, José I Cuende, Jose M de Miguel-Yanes, Eduardo Rovira, Demetrio Sánchez-Fuentes, Carmen Suárez Fernández, Pilar Román Sánchez, the SEMI Working Group

**Affiliations:** Hospital Regional Universitario, IBIMA, Málaga, Spain; Cardiometabolic Risk Unit, Internal Medicine Department, Hospital Virgen de los Lirios, Alcoy, Alicante, Spain; Internal Medicine Department, Hospital Carlos III, Madrid, Spain; Cardiovascular Risk Unit, Internal Medicine Department, Complejo Asistencial Universitario de Palencia, Palencia, Spain; Internal Medicine Department, Hospital Universitario del Sureste, Madrid, Spain; Internal Medicine Department, Hospital Universitario La Ribera, Alzira, Valencia, Spain; Internal Medicine Department, Hospital Nuestra Señora de Sonsoles, Ávila, Spain; Internal Medicine Department, Hospital Universitario de La Princesa, Madrid, Spain; Internal Medicine Department, Hospital General de Requena, Valencia, Spain

**Keywords:** Elderly patients, Cardio-metabolic risk factors, Cardiovascular diseases

## Abstract

**Background:**

Despite the progressive increase in life expectancy and the relationship between aging with multi-morbidities and the increased use of healthcare resources, current clinical practice guidelines (CPG) on cardiometabolic risk cannot be adequately applied to elderly subjects with multiple chronic conditions. Its management frequently becomes complicated by both, an excessive use of medications that may lead to overtreatment, drug interactions and increased toxicity, and errors in dosage and non-compliance. Concerned by this gap, the Spanish Society of Internal Medicine created a group of independent experts on cardiometabolic risk who discussed what they considered to be unanswered questions in the management of elderly patients.

**Discussion:**

Current guidelines do not specifically address the problem of elderly with multiple chronic conditions. For this reason, the combined use of the limited available evidence, clinical experience and common sense, could all help us to address this unmet need. In very old people, life expectancy and functionality are the most important factors for guiding potential treatments. Their higher propensity to develop serious adverse events and their shorter lifespan could prevent them from obtaining the potential benefits of the interventions administered.

**Summary:**

In this document, experts on cardiometabolic risk factors have established a number of consensual recommendations that have taken into account international guidelines and clinical experience, and have also considered the more effective use of healthcare resources. This document is intended to provide general recommendations for clinicians and to promote the effective use of procedures and medications.

## Background

In the last years, life expectancy has increased greatly worldwide. It is estimated that in 2050 the percentage of individuals over 65 years will represent 27% of the population of the European Union [1], and nearly 30% in Spain [2]. If the current aged-based decrease in the mortality rate is maintained in Spain, life expectancy at birth could reach 84.3 and 89.9 years in males and females, respectively by 2048 [2].

Chronic diseases increase with age. According to the Spanish version of the European Health Interview Survey (EHIS), 86% of subjects aged 75 or above declare to have at least one chronic clinical condition [3]. Age and chronic diseases are closely related to multi-morbidity which in turn, associates with an increased mortality, a decreased functional status, a reduced quality of life, and an increased utilization of healthcare resources [4].

Cardiovascular disease (CVD) is the most prevalent disorder in subjects with various comorbid conditions. In the PROFUND project, designed to develop a predictive model for patients with multi-morbidity, heart diseases were present in 78% of the study population at baseline. Moreover, cardiovascular risk factors such as hypertension, obesity, diabetes and dyslipidemia, were reported among the most frequent comorbidities (71.8, 70.5, 45.6 and 28.9% respectively) [5, 6]. In fact, CVD remains the main cause of death at older ages [7, 8].

Clinical Practice Guidelines (CPG) have been developed to help clinicians to improve the management and quality of care of specific diseases. However, at present, most CPG cannot be adequately applied to older patients with multiple chronic conditions. They are mostly based on clinical data provided by trials that have included either a small number of elderly patients or none at all [7]. Moreover, studies in people older than 80 years are scarce, despite the fact that they represent an increasing population with a different clinical profile compared with old under 80. Furthermore, CPG generally provide guidance for managing single conditions, but they rarely give advice on various chronic conditions at a time [9]. Aware of these limitations, a comprehensive statement for the secondary prevention of CVD in older adults has been recently published [10]. It is also striking that functionality is usually neglected, either in clinical studies or in guidelines, despite being a main issue in the elderly care.

For all these reasons, the management of the elderly is difficult, frequently complicated by an excessive use of medications that may lead to overtreatment, increased toxicity and drug interactions, as well as by errors in dosage and non-compliance [11]. After considering the aforementioned factors, and within the framework of the “Spanish National Health System (SNS) strategy” for approaching chronicity [12], the Spanish Society of Internal Medicine (SEMI) has coordinated this expert panel to analyse some relevant questions related to the management of the cardio-metabolic risk in elderly patients. A previous panel was created in November 2012 to address the cardiovascular risk in the general population [13]. In this occasion the experts have been asked to cover this same topic but focused on the elderly, with the aim of providing a better approach to help clinicians in their daily practice. With this aim, the Clinical Coordinators of the various Working Groups of the Spanish Society of Internal Medicine were asked to select 16 specific questions that they considered unresolved or that could cause uncertainty, in relation to the management of dyslipidemia; diabetes, obesity and nutrition; high blood pressure and antiplatelet therapy in the elderly, four questions for each topic. After a detailed analysis of the medical literature, a total of 17 questions (5 related with the antiplatelet therapy) were initially selected for discussion. During the debate to reach a consensus, one question related with diabetes, obesity and nutrition topic was discarded, leaving a total of 16 questions.

These questions were proposed for discussion to 59 participants (15 participants for each topic), previously selected for their extensive knowledge on the topic under discussion. Participants received relevant bibliography related to each question in advance which was previously selected by the specific Coordinator. All questions were discussed for approximately two hours and answered by consensus.

We must underscore that this manuscript is not a systematic review, but rather a statement based on the opinions of experts.

## Discussion

The answers to each question are presented here:

### Dyslipidemia

#### Are vascular risk equations useful for estimating the cardiovascular risk in the elderly Spanish population?

The vascular risk estimation in the elderly must be addressed by a comprehensive evaluation, including other important considerations (function, frailty, cognitive state, quality of life and life expectancy).

There is a lack of large cohorts aimed to estimate the cardiovascular risk of the Spanish population [14]. For this reason, the SCORE [14] and the Framingham [15] risk equations have been calibrated to be used in Spain [16, 17]. Both SCORE and Framingham risk equations, similarly classify high risk individuals. Nevertheless, and as suggested by other Spanish Societies, when vascular risk needs to be estimated the initial use of the adapted SCORE chart is recommended. Since the age limit established for estimating the risk in the SCORE chart is 65 years, older subjects may be allocated in this maximum age, even assuming that their risk could be underestimated. If the estimated risk is not high according to the SCORE chart, we recommend the use of the calibrated Framingham risk equation in order to avoid missing the few patients that could be reclassified as high risk according to this chart. The adapted Framingham risk equation allows calculations up to 79 years of age, although we admit it is not adequate to predict cardiovascular events in subjects over 80 years of age [18]. We also admit that there is an urgent need to develop national risk equations that could be applied to subjects of all ages. In the meantime, and considering the need of estimating the risk in older individuals, we should use the available risk charts with the chance of risk underestimation.

Cardiovascular risk scores should never replace clinical judgment, especially in older patients. An individualized decision-making process based on functional and cognitive status, quality of life and life expectancy, comorbidities and the patient’s preferences is critical in the elderly. Elderly-specific validated scales that include relevant markers for this population such as frailty and disability, have to be developed [19].

#### Should all the elderly be considered a high risk population?

Due to the inability of risk charts to predict risk in the elderly, it should be asked whether age could be considered a risk factor by itself and, therefore, prompt the initiation of preventive strategies. In fact, in the case of elderly patients without known CVD, classical risk factors do not accurately identify high risk individuals [18]. Although age is an accepted independent cardiovascular risk factor, not even very elderly subjects should be categorized as high risk individuals just for their age. Thus cardiovascular risk evaluation should be recommended to all age groups [20].

#### Is statin treatment useful for the primary prevention of CVD in the elderly?

There are few studies evaluating the efficacy of statins in the primary prevention of CVD in the elderly. Life expectancy in Spain at 80 years is approximately 5–6 years in males and 8 years in females, a sufficient time frame for obtaining a benefit from statins. In a meta-analysis of eight randomized trials by Savarese et al. [21], comparing statin treatment vs placebo in subjects over 65 years without established CVD, statins significantly reduced the incidence of myocardial infarction (MI) and stroke, although survival was not significantly prolonged. However, it must be underscored that this meta-analysis did not give information about patients older than 80, and the numbers needed to treat were relatively high (83 for MI and 142 for stroke).

The panel agreed that the use of statins in primary prevention may be beneficial, although after considering the evidence and cost-benefit reasons, they should be exclusively recommended to high-risk individuals with good functionality.

#### Is statin treatment useful for the secondary prevention of CVD in the elderly?

The evidence of cardiovascular risk reduction with statin treatment in secondary prevention is clear. Several reports have addressed a similar treatment benefit both in the elderly and in younger patients [22]. Statins reduce the risk of MI and stroke, as well as all-cause mortality in patients over 65 years of age [23, 24]. Considering the evidence, and in accordance with the European recommendations [25], we agree that statins treatment should be identically considered in both elderly and younger patients with established CVD.

### Diabetes, obesity and nutrition

#### What would be the recommended glycemic target in elderly Spanish diabetic patients?

Recent recommendations point out the need of a tailored antidiabetic treatment for older diabetics. When making treatment decisions, this approach takes into consideration, the history of hypoglycemias, comorbidities, self-care abilities, cognitive decline, functional status, and life expectancy [26]. Avoiding hypoglycemias is critical in this population, since elderly patients are at an increased risk of hypoglycemia, often the unaware type, which may have serious consequences (falls and functional and cognitive impairment) [27]. Several guidelines about the treatment of type 2 diabetes in the elderly have been recently published [28, 29]. Accordingly, the consensus emphasizes the importance of establishing a less tight control of glycemic parameters, balancing the risk/benefit of treatment, and considering the patient general status [30]. Renal and hepatic impairment and potential drug interactions should be also taken into account. The use of sulphonilureas should be restricted in the elderly, favoring the use of antidiabetic drugs with a low risk of hypoglycemia (metformin, DPP-4 inhibitors) [30]. Initiatives such as those of the Institute for Diabetes in Older People (IDOP) (http://instituteofdiabetes.org) can be useful for increasing the quality of care in this population, including diabetes care at home.

#### Is there any evidence that intentional weight loss could be beneficial for overweight/obese elderly diabetic patients?

Diet and exercise are important components for optimizing both general and cardiovascular health in older adults. Weight loss is generally recommended for reducing comorbid conditions. However, this recommendation may not be suitable for old patients. On one hand, sarcopenic obesity is highly prevalent in the elderly [31]. On the other hand, the obesity paradox [32] reminds us that someone who has achieved old age probably shouldn’t be encouraged to lose weight. Moreover, in the elderly, weight loss is associated with a high risk of bone loss related to energy restriction, and decreased nutrient intake. Therefore, very restrictive diets must be avoided in the elderly. Protein intake should be at least 0.8 g/kg body weight unless there was severe renal impairment [33]. The Mediterranean diet gives an adequate dietary fiber amount and low saturated fats, and has shown favorable effects on cardiovascular risk factors and outcomes in older adults [34]. Vitamin D deficiency is also very common in this population [35] and has been identified as an independent risk factor for cardiovascular mortality in older adults [36]. Hence, the strategy for this population should be to recommend a combination of physical activity and nutritional therapy, including a higher intake of calcium, vitamin D and other nutrients [37].

Increasing physical activity in older adults should reduce sedentary behavior and emphasize moderate-intensity aerobic activity. The activity plan must take into account the older adult’s abilities and aerobic fitness. Activities aimed to increase flexibility, muscle-strengthening and balance are also recommended [38].

#### Is nutritional assessment necessary in hospitalized elderly diabetic patients?

Malnutrition is an important problem in the ageing population with potential serious consequences. Several studies have found a relationship between malnutrition and, prolonged hospitalization, increased costs, and higher number of readmissions. Therefore, early detection and management of malnutrition is crucial in order to prevent future complications [39]. A number of screening tools are already available to assess nutritional status. Although not specifically focused on geriatric people, they can be administered to the elderly because of their simplicity, speed and easy design [40].

### High blood pressure

#### Which blood pressure targets should be applied to the elderly population?

It is difficult to make recommendations about how to manage high blood pressure (BP) in older adults. Most of the information has been largely obtained from studies of isolated systolic hypertension and only one study has included people over 80 years [41]. On the other hand, the age-related increase in systolic BP could be considered a physiological phenomenon that usually goes together with a diastolic BP decrease [42]. Various meta-analyses confirmed a differential effect of antihypertensive drugs on morbidity and mortality in relation to age (65–79 vs. 80 or older). In subjects older than 79 years, antihypertensive drugs reduce nonfatal vascular events (mainly strokes), but increase total mortality when the diastolic BP decrease below 70 mmHg [43, 44]. Some epidemiological studies showed a J shaped relationship between BP and the incidence of cardiovascular events [45], with different systolic and diastolic BP nadir values when comparing subjects between 65–79 years of age with those aged 80 years or older [46].

It is acknowledged that for hypertensive subjects aged 65 to 79 years, the evidence obtained from younger adults should be applied. However, that should not be the case for people over 79 years, where there is no evidence, neither from clinical trials nor from meta-analysis, of the optimum blood pressure targets. It is also acknowledged that BP targets must be established according to the functional status, not just the age, and that a general BP target could be 140/90 mmHg, always avoiding a diastolic BP lower than 75–80 mmHg.

#### Which should be the first drug to be use in elderly hypertensive patients?

There is a lack of studies comparing various antihypertensive drugs in older individuals. Although the beneficial effects of the antihypertensive drugs shown in the meta-analysis are similar to the ones obtained in younger adults, it should be emphasized that no previous meta-analysis has included people older than 80 years, or considered their frailty/functional status [47].

Taking all these aspects into consideration, angiotensin-converting-enzyme inhibitors, calcium channel blockers and their fixed combinations, should probably be the best pharmacological options due to their better tolerability. Initial lower doses and a slower titration should be recommended in elderly patients in order to avoid adverse events and a negative impact on quality of life and functionality.

#### Beyond blood pressure, which aspects should be considered when making decisions about antihypertensive treatment in elderly patients?

Most clinical guidelines acknowledged that, in many aspects, there is a lack of evidence on hypertension management in the elderly. They usually point out that when speaking about elder individuals, there are no proper guidelines but rather consensual documents. All these guidelines have also emphasized that functionality, comorbidity and life expectancy, are more relevant indicators for choosing a treatment than just age [48–50]. A recent study has shown that for an elderly hypertensive population, frailty is one of the strongest predictors of mortality [51]. Despite this, there is no specific trial on antihypertensive treatment focusing on functional status rather than on age.

#### Would ambulatory blood pressure monitoring (ABMP) have a specific role in elderly hypertensive patients?

ABPM could be specially indicated in this population. In the very old, as showed in the HYVET substudy, white coat hypertension is present in at least 50% of subjects [52]. Due to this high prevalence of white coat hypertension and to the risk of diastolic hypotension when trying to control systolic BP, ABPM should be specially considered to ensure diagnosis and for an accurate individualization of pharmacological treatment [53].

### Antiplatelet therapy

#### When is antiplatelet therapy indicated in elderly patients without CVD?

The use of aspirin in primary prevention confers a modest benefit in nonfatal MI and CVD events and greatly increases the risk of bleeding associated with aspirin. For this reason, the risk/benefit ratio for recommending its use, should be carefully evaluated on a patient by patient basis [54].

#### Is antiplatelet therapy a more reasonable (risk/benefit) alternative than anticoagulation in elderly patients with atrial fibrillation?

Overall, anticoagulation (with anti vitamin K or new oral anticoagulants) provide a net benefit over antiplatelet therapy in elderly patients with atrial fibrillation. One possible exception, due to lack of evidence, would be patients with CHADS-VASc = 1, especially if they have a high bleeding risk (HAS-BLED > =3).

Due to the higher risk of major bleeding associated with anticoagulation, the selection of antithrombotic therapy in this population should balance the risk for embolism and the risk for hemorrhage. In this setting the use of CHADs-VASC and HAS-BLEED scores is mandatory. The net clinical benefit is higher in subjects with a higher risk of stroke, including the oldest age category, despite their higher bleeding risk [55].

#### Is there any indication for dual antiplatelet therapy in the elderly?

Dual antiplatelet therapy, usually aspirin and clopidogrel, is a general recommendation in patients with an acute coronary syndrome and after percutaneous coronary interventions [56, 57]. In the CURE trial [58], the addition of clopidogrel to aspirin showed similar absolute reductions (although smaller relative reductions) in the combined ischemic end points, in older as compared to younger patients.

#### Is there any indication for both antiplatelet therapy and anticoagulation in the elderly?

The addition of antiplatelet therapy to anticoagulants in older patients, is indicated in the same clinical conditions that are recommended in younger individuals (such as atrial fibrillation plus coronary stent placement), always taking into account the higher risk of bleeding at this age [57].

#### Does aging involve any exceptions regarding the general recommendations on antiplatelet therapy in CVD?

In general, aging alone is not a contraindication to follow the general recommendations on antiplatelet therapy in CVDs, although treatment decisions should always consider life expectancy and functional status.

The evaluation of both CHADS-VASc and HAS-BLED is very important in the elderly, although patients with a high CHADs-VASC usually have also a high HAS-BLED.

## Summary

There is a general perception of a gap in the current cardiovascular guidelines, which do not specifically address the problem of the elderly with multiple chronic conditions. For this reason, interpretation of the scarce available evidence, clinical experience and common sense, should be taken into consideration when addressing this unmet need. For very old people, life expectancy as well as functionality are the most important issues to be considered, mainly because older subjects are prone to frequent adverse events and their lifespan could prevent them from achieving the potential benefits of the interventions administered.

This document provides some consensual recommendations about general unanswered questions in cardiometabolic risk in the elderly. International guidelines and clinical experience have been considered as well as the most effective use of health care resources. Far from trying to increase the available information, this document is intended to provide general recommendations for clinicians and to promote the effective use of procedures and medications. We must underscore that this manuscript is not a systematic review, but rather a statement based on the opinions of experts.

## Abbreviations

EHIS: European health interview survey
CVD: Cardiovascular disease
CPG: Clinical practice guidelines
SNS: Spanish national health system
SEMI: Spanish society of internal medicine
BP: Blood pressure
ABPM: Ambulatory blood pressure monitoring
MI: Myocardial infarction
